# Association between mild stimulated IVF/M cycle and early embryo arrest in sub fertile women with/without PCOS

**DOI:** 10.1186/s12958-020-00622-y

**Published:** 2020-07-15

**Authors:** Nagwa Elshewy, Dongmei Ji, Zhiguo Zhang, Dawei Chen, Beili Chen, Rufeng Xue, Huan Wu, Jianye Wang, Ping Zhou, Yunxia Cao

**Affiliations:** 1 Reproductive Medicine Center, Department of Obstetrics and Gynecology, the First Affiliated Hospital of Anhui Medical University, Hefei, China; 2 Key Laboratory of Population Health Across Life Cycle (Anhui Medical University), Ministry of Education of the People’s Republic of China, Hefei, China; 3 Anhui Province Key Laboratory of Reproductive Health and Genetics, Hefei, China; 4 NHC Key Laboratory of Study on Abnormal Gametes and Reproductive Tract, Anhui Medical University, Hefei, China; 5 Anhui Provincial Engineering Technology Research center for Bio preservation and Artificial Organs, Hefei, China

**Keywords:** IVM, Embryo arrest, Mitochondria, Aneuploidy

## Abstract

**Background:**

The in vitro maturation (IVM) technique has physical and financial benefits, but a lower efficiency and outcome that is still unclear whether it is related to polycystic ovary syndrome (PCOS) itself or the IVM procedure. In this study, we analyzed the clinical and laboratory outcomes of an optimized IVM protocol in patients with and without PCOS. We also discussed the possible reasons for early embryo arrest in the IVM cycle.

**Methods:**

This prospective study involved 58 PCOS patients and 56 non-PCOS patients who underwent mild stimulated IVF combined IVM (IVF/M) cycles. The clinical and laboratory outcomes were compared between the two groups. Also, metaphase II (MII) oocytes were obtained after IVM from the two groups, and in vivo MII oocytes randomly collected from IVF patients were examined for mitochondrial function using a laser scanning confocal microscope (LSCM). The aneuploidy rate for arrested cleavage embryos from IVM and IVF oocytes were screened using Next Generation Sequencing (NGS).

**Results:**

Mildly stimulated IVF/M resulted in cumulative clinical pregnancy and implantation rates (40.2, 28.7% in the PCOS group vs. 41.9, 36% in the non-PCOS group), respectively. The blastocyst formation rates were comparable (28% vs. 28.2%) in PCOS and non-PCOS groups, respectively. Using LSCM, there was a significant decrease in the mitochondrial membrane potential of IVM oocytes compared with the control IVF oocytes (*P* < 0.001), but no significant difference between the PCOS and non-PCOS groups. The NGS showed that the aneuploidy rates were comparable (75, 75, and 66.6%) in IVM-PCOS, IVM-non-PCOS, and control IVF arrested embryos, respectively.

**Conclusions:**

The mildly stimulated IVF/M protocol produced acceptable clinical outcomes in PCOS and non-PCOS patients. IVM itself rather than the PCOS condition adversely affected the embryo development through its effect on mitochondrial function, which appeared to be a possible cause for the embryo arrest in the IVM cycles rather than chromosomal aneuploidy.

## Introduction

In vitro maturation of human oocytes (IVM) is a promising laboratory technique in which germinal vesicle (GV) and metaphase I (MI) oocytes proceed to metaphase II (MII) oocytes. Both immature and mature oocytes can be retrieved in natural cycles or mildly stimulated cycles using hCG or GnRH agonist priming. Therefore, the procedure is better defined as the IVF cycle combined with IVM [1].

While a conventional belief exists that the growth of a dominant follicle suppresses the growth of the remaining smaller follicles that leads to their atresia, natural cycle IVF combined with IVM (IVF/M) has shown that these enclosed, arrested oocytes can resume meiosis and complete maturation in the lab. Natural cycle IVF/M has achieved higher cumulative clinical pregnancy rates with in-vivo mature oocytes obtained from dominant follicles and immature oocytes retrieved from smaller follicles after IVM culture [2]. The Lim-Chian protocol for natural cycle IVF/M has shown that injection of 10,000 IU hCG could avoid premature ovulation while the dominant follicle was at a suitable size (diameter between 12 and 14 mm) [3]. Natural cycle IVF/M has been an attractive choice for infertility treatment for various reasons. However, it resulted in fewer numbers of retrieved oocytes due to small size in addition to the in vitro maturation culture defect.

Therefore, researchers have implemented various protocols to improve the outcome of IVM, including unstimulated, FSH priming, hCG priming, and FSH combined hCG priming. However, as described above, most IVM protocols used FSH priming to promote follicular growth, while the use of hCG triggering was controversial. Several embryological studies reported that the use of 10,000 IU hCG injection before oocyte retrieval during an IVM cycle could hasten oocyte maturation in vitro and improve the maturation and developmental competence of oocytes [4–8]. However, others believed that the retrieval of mature oocytes with hCG triggered in IVM cycles was contrary to the basis of that procedure [9, 10]. In addition, there was no definitive evidence for the effect of hCG triggering on clinical pregnancy or miscarriage rates, and live birth rate in IVM cycles [11].

Despite the advanced improvements in clinical IVM protocols that have occurred and the fact that the IVM culture media resulted in satisfactory clinical pregnancy rates comparable with standard IVF protocols in the well-selected patients [9, 12–14], IVM cycles showed a lower blastocyst formation rate compared to standard IVF cycles [15]. Furthermore, the biological loss of oocytes during in vitro maturation culture resulted in lower chances of pregnancy for some patients.

In comparison to IVF, which can achieve 40–70% blastocysts and others that arrest at different earlier stages [16–18], blastocyst embryo formation is observed to be only 20% from IVM cycles [12]. This lower percentage reflects the higher incidence of early embryo arrest in IVM cycles. Previous researchers attributed the early embryo arrest to multiple factors, including parental genetic causes, oxidative stress [19], energy stores defects [20], and aneuploidy [21].

Despite the fact that IVM is mainly indicated for patients with polycystic ovary syndrome (PCOS), it remains unclear whether the low efficiency is due to the IVM procedure or PCOS of a combination of the two factors. In the current study, we compared the clinical and laboratory outcomes of mildly stimulated IVF combined with IVM (IVF/M) in PCOS and non-PCOS patients. Furthermore, we evaluated the efficiency of the IVM technique with respect to the in vitro maturation rate, fertilization rate, cleavage embryo rate, and blastocyst formation rate for both groups.

To unravel the underlying mechanism of early embryo arrest, we used laser scanning confocal microscope (LSCM) to measure the impact of IVM culture on mitochondrial membrane potential (ΔΨm), which forms the basis of energy metabolism in cells, in MII oocytes matured in vitro in PCOS and non-PCOS groups compared to randomly collected in vivo MII oocytes. In addition, we used Next-Generation Sequencing (NGS) to determine the aneuploidy rates in arrested (IVM and IVF) embryos.

## Materials and methods

### Patients

This prospective study included 114 patients who were examined for mildly stimulated IVF/M cycles from September 2018 to November 2019 at the reproductive medical center, in the first affiliated Hospital of Anhui Medical University, Hefei, Anhui Province, China. The patients, aged < 35 years old, had normal basal FSH levels (< 10 mIU/mL) and a body mass index (BMI) range of 19–25 kg/m2. Patients with normal ovulatory or anovulatory cycles with any cause for infertility, including tubal factors, mild to moderate male factors, and unexplained infertility, were enrolled in this study. Patients were divided into two treatment groups. Group A included anovulatory patients diagnosed as PCOS according to Rotterdam criteria [22], and Group B included ovulatory non-PCOS patients.

On day 3 of their menstrual cycle, blood samples were obtained for endocrine tests for FSH, LH, prolactin, testosterone, and estradiol. All patients were assessed for metabolic syndrome by measuring total cholesterol, HDL, LDL cholesterol, triglycerides, and their level of insulin-resistance using fasting insulin and fasting glucose tests.

Patients who donated MII oocytes during COH/IVF cycles were chosen randomly to serve as a control group in the investigation of ΔΨm under confocal microscope. These patients, aged < 35 years old (29 ± 2.5), had an average duration of infertility of 2 (± 1.5) years, BMI of 22 (± 1.6) kg/m2, FSH level of 6 (± 1.2) mIU/mL and a LH level of 4 (± 1.6) mIU/mL. Nine MII oocytes were obtained from eight patients. Of these, two patients were diagnosed with PCOS, four patients with tubal factor infertility and two patients with unexplained infertility and normal ovarian function.

### Mildly stimulated IVF/M protocol

The treatment cycle was initiated based on an ultrasound scan on day 3 of a spontaneous or a progestin-induced withdrawal bleeding. All participants had a total of 7 or more antral follicles in each ovary. A short course of gonadotropins with an initial dose of 150 IU of r-FSH (Gonal-F, Merck co, Switzerland) was administered for 4–5 days, beginning on cycle Day 3 or 4, when the follicle size was 5 or less mm in diameter. The follicle diameter and endometrium thickness were assessed. Oocyte retrieval was scheduled when the follicle reached 10–12 mm in diameter when an hCG injection of 10,000 IU (Ovidrel, Merck-Serono, U.K) was applied 36 h before oocyte retrieval.

### Oocyte collection, IVM, sperm preparation, and ICSI

Oocyte retrieval was performed using transvaginal ultrasound–guided aspiration with a 17-gauge single-lumen needle (Cook, Eight Mile Plains, Australia), connected to a portable pump with a pressure of less than 90 mmHg. Oocytes were examined on the day of aspiration for maturity after denudation of cumulus cells. The mature oocytes were inseminated using intracytoplasmic sperm injection (ICSI) on the day of oocyte retrieval. The immature oocytes were cultured for 24–48 h in IVM medium prepared in the IVF lab of our reproductive medical center. The IVM medium consisted of 80% IVM maturation medium, TCM-199 supplemented with 20% of the patient’s own serum, 0.05 IU/ml r- FSH, 0.05 IU/mL HCG, 0.04 mg/ml 17 B-estradiol, 0.2 mM/L pyruvic acid, and 0.05 ml of melatonin. The oocytes were cultured at 37 °C in 6% CO2, 5%O2, and 89% N2 with high humidity to allow for maturation.

The IVM culture medium consisted of a basic culture media, exogenous hormonal additives, and a source of protein. In particular, the maternal serum contains valuable components, including growth factors, amino acids, and some hormones that are essential for oocyte maturation. Moreover, these endogenous hormones can help promote oocyte maturation. In addition, PCOS patients (with a non-hyperandrogenic phenotype) recruited to our study had comparable endogenous hormonal levels with non-PCOS patients and no significant differences between the two groups of patients. Therefore, the addition of different sera did not interfere with the results of the in vitro culture of immature oocytes for the two groups.

After 24 h, we checked for the presence of a polar body in IVM oocytes. ICSI was performed for the in-vitro matured oocytes, and any immature oocytes remained in IVM media for another 24 h. Fresh sperm preparations were obtained twice on the day of ICSI for in vivo, and in vitro mature oocytes and ICSI was performed as a common routine in the IVF lab.

### Endometrial preparation and embryo transfer

On the day of oocyte collection, 4 mg estradiol valerate (Progynova, Jenapharm Gmbh, Germany) was administered twice daily for endometrial preparation for patients undergoing fresh embryo transfer (ET) with an endometrium thickness 8 mm or greater. Sixty mg progesterone in oil (Zhejiang Xianju Pharmaceuticals, china) was injected daily for luteal support starting on the day of ICSI. Two good quality cleavage embryos (more than 8 uniform cells and less than 20% fragmentation) were transferred on day 3 after insemination of the in-vitro matured oocytes. The embryos from the in-vivo matured oocytes and the remaining embryos from the in vitro matured oocytes were cultured to the blastocyst stage until day 5 or 6, and then the good-quality embryos were cryopreserved. Two weeks after ET, the serum β-hCG level was tested to detect pregnancy. Two weeks later, clinical pregnancy was confirmed by the appearance of a gestational sac with a fetal pole using ultrasound**.**

### Analysis of oocytes’ mitochondria under LSCM

A laser scanning confocal microscope (LSCM) was used to detect the impact of IVM culture on the mitochondrial membrane potential (ΔΨm). Three groups of oocytes were tested as follows: Group A, 7 MII oocytes after IVM from 5 PCOS patients, Group B, 7 MII oocytes after IVM from 6 non-PCOS patients, and Group C, (control) 9 MII oocytes randomly collected from 8 IVF patients less than 35 years of age. The MII oocytes examined in the IVM groups were matured in vitro after 24 h from GV oocytes.

All oocytes in the three groups showed normal morphology and were stained using the ΔΨm specific probe JC-1 (beyotime, C-2005), which was diluted to a final concentration of 5 μg/ml in embryo culture medium (G-1 + 5%HSA, Vitro life). After washing the oocytes twice with PBS, we added 0.5 mL of the staining solution to the oocytes and incubated them in a humidified incubator containing 6% CO2 at 37 °C for 15–30 min in the dark. Then, the oocytes were washed twice with 1XPBS and imaged with LSCM using the fluorescein isothiocyanate (FITC, green) and rhodamine isothiocyanate (RITC, red) channels. The captured images (green and red) were acquired in the largest diameter plane of the oocytes using the confocal software. The ratio of RITC to FITC for each oocyte was calculated and used as the endpoint for the ΔΨm [23].

### Next generation sequencing of arrested embryos

Cleavage was assessed after 72 h post ICSI. Arrested cleaved embryos that were unable to develop to the blastocyst stage on day 5, showed good quality and normal morphology with more than 4 cells in cleavage stage and obtained from zygotes with two pronuclei, were analyzed with NGS screening for whole chromosomes. Three groups of samples were analyzed: Group A, 12 arrested IVM embryos from 9 PCOS patients, Group B, 12 arrested IVM embryos from 10 non-PCOS patients, and Group C (control) 12 arrested IVF embryos randomly collected from 11 IVF patients less than 35 years old with the exclusion of ovarian insufficiency and male factor infertility that might be associated with paternal chromosomal abnormalities.

Briefly, the collected samples were washed in a PCR tube containing 2.5 μl sterilized 1XPBS. Whole genomic amplification (WGA) of the samples was performed using the Sureplex DNA Amplification System (Blue Gnome, Cambridge, UK). WGA products were quantified using the Quan Tit dsDNA HS Assay Kit (Life Technologies Corporation, Grand Island, NY, USA), as reported previously [24, 25]. Dual-indexed libraries were prepared using the Ion Xpress™ Plus Fragment Library Kit (# 4471269), the Ion Plus Fragment Library Kit (# 4471252), and Ion Xpress™ Barcode Adapters 1–96 with the input sample DNA at 1 μg gDNA (Illumina, San Diego, USA). Template-positive ISPs containing clonally amplified DNA were prepared by using the Ion PGM™ Template OneTouch™ 2200 Kit (# 4480974), and sequencing with the Ion PGM™ Sequencing 200 Kit v2 (# 4482006), Paired end, dual index 2x36bp sequencing was performed using the Ion Torrent Proton (Life Technologies Corporation, Grand Island, USA).

### Statistical analysis

Statistical analysis was performed using the SPSS version 21.0 software (SPSS, Chicago, IL). Differences between two groups were analyzed using unpaired t-tests. ANOVA was used for the analysis of multiple groups. Qualitative data were analyzed using the chi-square (χ^2^) test. Data were represented as the mean ± SD, and a *P*-value < 0.05 was considered statistically significant.

## Results

Aneuploidy or chromosome abnormalities are the leading causes for embryo arrest, recurrent implantation failure, recurrent miscarriage, and birth defects that appear to be age-associated, and about 30–60% of embryos are reported to be aneuploid in patients older than 35 years of age [26–29]. To exclude age-associated embryo arrest, we selected patients who were less than 35 years old.

According to the Rotterdam criteria for phenotypes of PCOS patients [22], all PCOS patients recruited in our study were categorized as the non-hyperandrogenic PCOS phenotype (olig-anovulation and polycystic ovaries morphology) with normal endocrine test results and metabolic profiles. Patients with male factor infertility exhibited mild to moderate oligospermia (count > 5 M/ml), asthenospermia (progressive motility < 32%), and abnormal morphology < 96%. All patients with male infertility underwent screened chromosome analysis to exclude genetic abnormalities.

We did not observe any significant differences related to age, BMI, FSH levels on day 3 of the cycle, and duration of gonadotropin administrations between PCOS and non-PCOS patients as shown in Table 1.

**Table 1:** Characteristics of patients enrolled in our study

Groups	A (PCOS)	B (non-PCOS)	*P* value
No of patients	58	56	
Age	28.0 ± 3.26	28.0 ± 3.64	NS
Body mass index (BMI) kg/m2	23.0 ± 2.4	22.0 ± 1.7	NS
FSH level	5.0 ± 1.2	6.0 ± 1.5	NS
Days of gonadotropin stimulation	4.0 ± 1.5	4.0 ± 1.9	NS
Tubal factor infertility	35	37	-
Male factor infertility	8	6	-
Tubal and male factor infertility	5	5	-
Unexplained infertility	10	8	-

Data measured by (mean± SD), NS represent (*P*-value > 0.05)

Data measured by (mean ± SD), NS indicates a *P*-value > 0.05.

A total of 1375 oocytes were collected from all cycles from Group A (PCOS patients) and Group B (non-PCOS patients). Of these, at the time of retrieval, 168 MII (22.8%), 473 GV (64.6%), and 93 MI (12.6%) oocytes were obtained from PCOS patients, and165 MII (25.7%), 396 GV (61.7%), and 80 MI (12.4%) oocytes were obtained from non-PCOS patients. At 24 h of culture, the results showed the maturation rate in vitro was 63.6% vs. 54.6%, with a total maturation rate of 68.9% vs. 58.8% in PCOS and non-PCOS groups, respectively.

There was no significant difference in the total rates of fertilization, cleavage, and blastocyst formation between the groups. When the efficiency of the IVM procedure was compared between the POCS and non-PCOS groups, the fertilization and blastocyst formation rates did not show significant differences between the two groups. However, the rate of oocyte in vitro maturation in Group A showed a small but significant difference (*P* = 0.04) compared to Group B (Table 2).

**Table 2:** Lab outcomes of mildly stimulated IVF/M cycle in Group A (PCOS) and Group B (non-PCOS) patients

Groups	A (PCOS) (*n*=58)	B (non-PCOS) (*n*=56)	*P* value
No. of mature oocytes retrieved (mean ± SD)	168 (2.9±5)	165 (2.8±4.1)	NS
No. of in vivo mature oocytes fertilized (mean ± SD)	126 (2.1±3.6)	128 (2.2±3.6)	NS
No. of in vivo mature oocytes cleaved (mean ± SD)	107 (1.8±3.2)	111 (1.9±3.1)	NS
No. of blastocyst from in vivo mature oocytes (mean ± SD) (%)	72 (1.2 ± 2.1) (57.1%)	80 (1.3 ± 2.2) (62.5%)	NS
No. of immature oocytes retrieved (mean ± SD)	566 (9±5.7)	476 (8±5.3)	NS
No. of oocytes matured in vitro (%)	390 (68.9%)	280 (58.8%)	0.04
No. of in vitro mature oocytes fertilized (mean ±SD) (%)	285 (4.6±3.1) (73%)	205 (3.5±2.9) (73.2%)	NS
No. of in vitro mature oocytes cleaved (mean ± SD)	226 (3.6±2.7)	152 (2.5±2.2)	NS
No. of blastocyst from in vitro mature oocytes (mean ± SD) (%)	80 (1.3±1.2) (28%)	58 (0.9±1.1) (28.2%)	NS
Total no. of oocytes matured (mean ± SD)	558 (9±5.7)	445 (7±4.7)	NS
Total no. of oocytes fertilized (%)	411 (73.6 %)	333 (74.8%)	NS
Total no. of zygote cleaved (%)	333 (81%)	263 (78.9%)	NS
Total no. of blastocyst (%)	152 (36.9 %)	138 (41.4%)	NS

Data measured by (mean± SD) and percentage (%), NS represent (*P*-value > 0.05)

Mildly stimulated IVF/M cycles provided the patients with the chance for embryo transfer obtained from IVM oocytes and others obtained from in vivo MII oocytes. When a mean of 2 or 3 embryos was transferred, it resulted in cumulative clinical pregnancy rates of 40.2% vs. 41.9% and implantation rates of 28.7% vs. 36% in PCOS and non-PCOS groups, respectively. A fresh transfer of two cleavage embryos with IVM was performed in 44 IVM cycles. In addition, the transfer of frozen and thawed IVM blastocyst embryos (ET) was performed in 69 IVM cycles. Further, frozen and thawed ET of blastocysts obtained from in-vivo MII oocytes were transferred in 45 cycles.

For IVM/ET cycles, however, there were no significant differences between PCOS and non-PCOS patients with respect to fresh and frozen ET cycles. Thus, frozen and thawed ET cycles had a better outcome with respect to the clinical pregnancy rate and implantation rate in the two groups when compared with fresh ET cycles.

Frozen and thawed ET cycles for blastocysts obtained from in vivo MII oocytes yielded clinical pregnancy rates of 52.3% vs. 50% and implantation rates of 44% vs. 48% in PCOS and non-PCOS groups, respectively. No significant differences were observed between the two groups for biochemical pregnancy rate, miscarriage rate, and live birth rate. Seventeen patients delivered 14 healthy boys, and 5 girls, and 43 pregnancies were still ongoing at the time of manuscript preparation (Table 3).

**Table 3:** Clinical outcome of mildly stimulated IVF/M cycle in Group A (PCOS) and Group B (non-PCOS) patients

Groups	A (PCOS) (*n*=58)	B (non-PCOS) (*n*=56)	*P* value
**No. of IVM/ET cycles**	56	57	NS
**Clinical pregnancy rate**			
Cumulative rate	(20/56) 35.7%	(22/57) 38.5%	NS
Fresh ET rate	(7/21) 33.3%	(6/23) 26.1%	NS
Frozen ET rate	(13/35) 37.1%	(16/34) 47.1%	NS
**No. of embryo transferred**	83	75	NS
**No. of embryo implanted**	20	24	NS
**Implantation rate**			
Cumulative rate	(20/83) 24.1%	(24/75) 32%	NS
Fresh ET rate	(7/37) 18.9%	(6/35) 17.1%*	NS
Frozen ET rate	(13/46) 28.2%	(18/40) 45%*	NS
**No. of IVF/ET cycles**	21	24	
Clinical pregnancy rate	(11/21) 52.3%	(12/24) 50%	NS
Implantation rate	(11/25) 44%	(12/25) 48%	NS
**Total no. of ET cycles**	77	81	
**Cumulative pregnancy rate**	(31/77) 40.2%	(34/81) 41.9%	NS
**Cumulative implantation rate**	(31/108) 28.7%	(36/100) 36 %	NS
**No. of biochemical pregnancy**	(33/77) (42.8%)	(35/81) (43.2%)	NS
**No. of miscarriage**	(3/33) (9%)	)2/35) (5.8%)	NS
**No. of livebirth**	9	10	NS
**No. of ongoing pregnancy**	20	23	

Data measured by percentage (%); NS represent (*P*-value > 0.05)

N.B: * represent significant difference between implantation rate of fresh and frozen ET in group B (*P* <0.05)

**Fig. 1:**
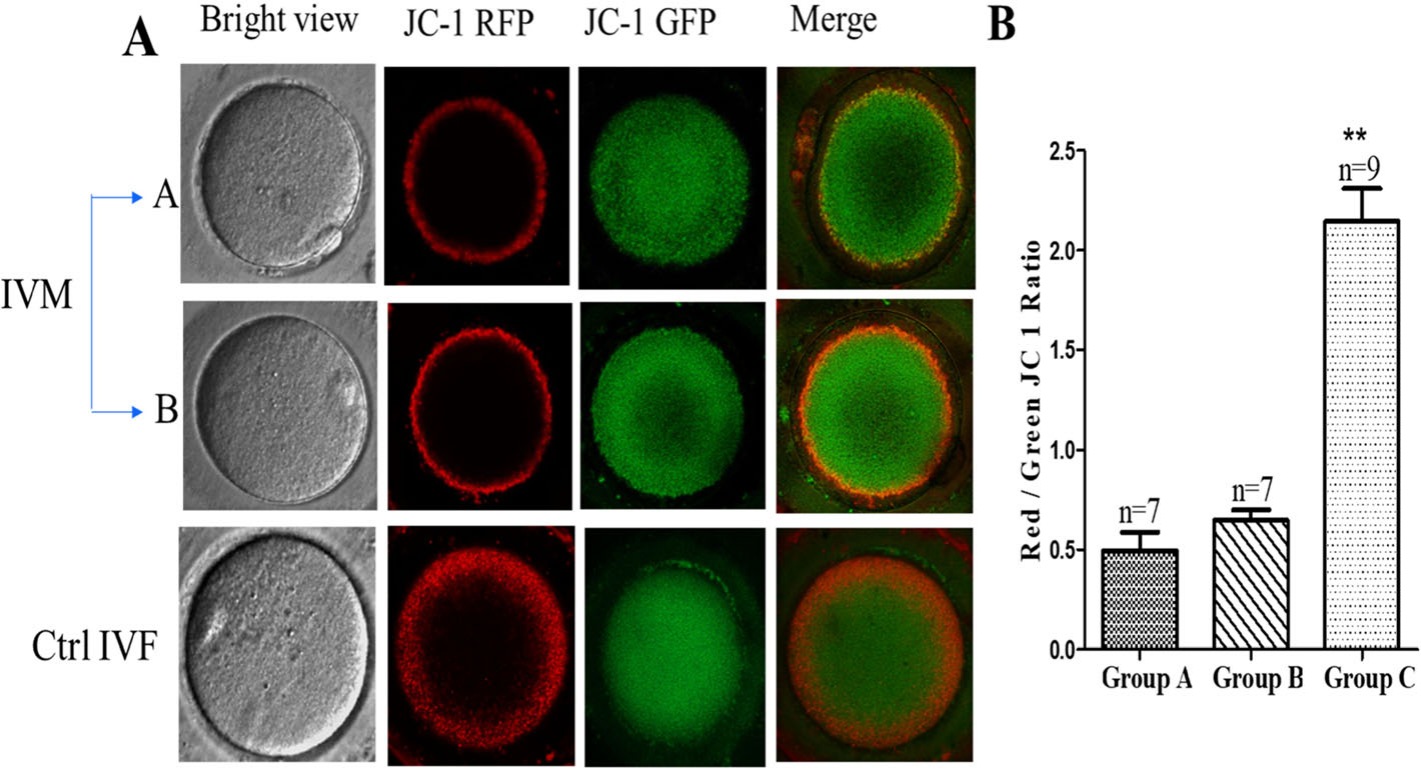
Effect of IVM on the inner mitochondrial membrane potential (ΔΨm) measured by JC-1 fluorescence, **a**: Relative inner membrane potential in MII oocytes from A: IVM-PCOS, B: IVM-non PCOS and C: control IVF groups. **b**: Ratio of red to green JC-1 fluorescence in the three groups. Data measured by mean± SD, ** represent *P* < 0.05

MII oocytes were stained with the mitochondria-specific probe, JC-1, and visualized with LSCM, a red fluorescent stained, which located around the periphery of the oocyte indicates higher mitochondrial membrane potential. While, green fluorescence is dispersed within the oocyte cytoplasm indicates lower mitochondrial membrane potential. We found that oocytes matured in vitro presented reduced red fluorescence in periphery of the cell compared to oocytes that were matured in vivo; the green fluorescence had no observable change (Fig. 1a). We investigated the high polarization of mitochondria in MII oocytes by examining the relative ratio of emitted red to green fluorescence. The ratio of red to green JC-1 fluorescence in MII oocytes from IVM patients sharply decreased, compared with the control group C (MII oocytes from IVF patients, *P* < 0.001), which indicated that the ΔΨm was decreased without differences between the IVM groups (PCOS and non-PCOS) (*P* = 0.7) as shown in Fig. 1b.

**Fig. 2:**
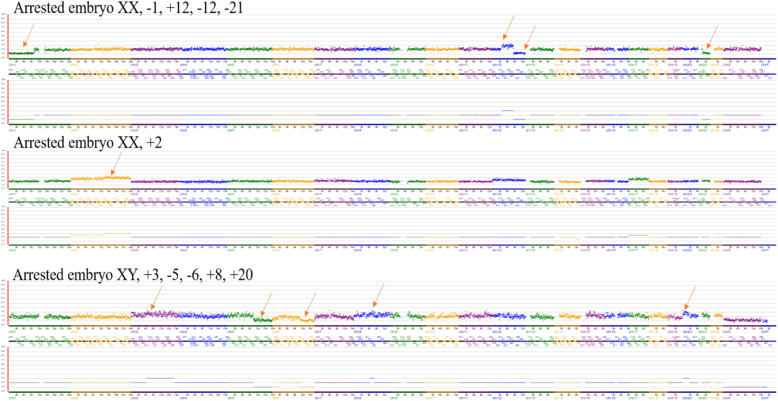
NGS charts of arrested embryos with different types of chromosomal abnormalities, Notes: Arrows indicate chromosome errors.

Next generation sequencing (NGS) is a recent technology used for preimplantation genetic diagnosis [30], and it was employed to analyze 36 arrested cleaved embryos obtained from our cycles **(**Fig. 2**)**. Our findings reveled that the aneuploidy rates in arrested embryos of IVM-PCOS, IVM-non PCOS, control IVF were (75, 75, and 66.6%), respectively, with no significant difference among the three groups as shown in Table 4. Furthermore, the different types of chromosomal abnormalities such as monosomy, dual, trisomy, complex, and mosaicism were observed in the three groups and suggested that aneuploidy is not the possible cause for embryo arrest.

**Table 4:**
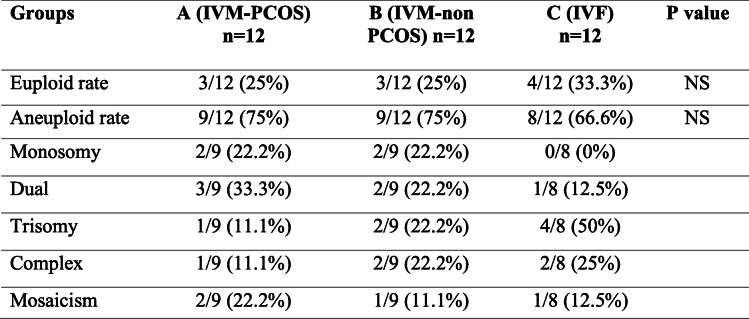
Next generation sequencing determinations for euploidy and aneuploidy in arrested embryos from IVM-PCOS, IVM-non-PCOS, and control IVF groups

Data measured by percentage (%); NS indicates *P* value > 0.05

## Discussion

Standard IVF is superior to IVM in terms of the clinical pregnancy rate, implantation rate, and number and quality of embryos. However, the IVM procedure has physical, physiological, and financial advantages. Although IVM is indicated to avoid ovarian hyper stimulation syndrome (OHSS) in PCOS patients and recently used for fertility preservation in cancer patients, it is still in its an underutilized procedure due to its low efficiency. It is still not known whether the low efficiency is due to the PCOS condition or the IVM itself.

To investigate the IVM efficiency, we administered FSH combined with hCG priming to improve the nuclear maturation rate and increase the number of retrieved mature oocytes compared to non-hCG primed cycles, while the FSH priming improved the competence of oocytes for proper development [31]. Our experience has shown that it is easier to retrieve more oocytes from follicles around 10 mm in diameter compared to smaller follicles.

In line with Lim et al. [3], mildly stimulated IVM cycles with FSH combined hCG priming resulted in 22.8 and 25.7% mature oocytes from small follicles less than 12 mm in diameter in PCOS and non-PCOS groups, respectively. This might confirm the presence of LH or hCG receptors in the cumulus and granulosa cells from the small follicles [32]. Even so, it is unclear how the small follicles react to the LH surge.

The non-hCG primed IVM cycles yielded a fewer number of retrieved mature oocytes compared to the hCG primed cycles. Researchers thought that these mature oocytes had completed the nuclear maturation while the cytoplasmic maturation was not completed. This lack of synchronization resulted in lower fertilization and blastocyst formation rates compared to standard IVF [14, 33]. However, in this study, the mature oocytes with FSH and hCG priming were capable of being fertilized (75, 77.5%) and developed to blastocyst embryos (57.1, 62.5%) in PCOS and non-PCOS groups, respectively, as shown in Table 2, and even the size of these follicles was less than the size in standard IVF protocols.

In line with Chian et al., the developmental competence of immature oocytes from small follicles was not impaired by the presence of mature follicles [2]. In addition, it was reported that the clinical pregnancy rate was higher in the IVF/M cycle with in vivo matured oocytes (40.0% vs. 23.3%) compared to the IVM cycles with immature oocytes only [3, 34]. These observations support our findings that mildly stimulated IVF/M cycles could achieve a higher cumulative clinical pregnancy rate and implantation rate (40.2, 28.7% in the PCOS group vs. 41.9, 36% in the non-PCOS group), respectively.

Most researchers believed that PCOS patients are the ideal group to undergo the IVM procedure due to a large number of antral follicles, oocyte retrieved, and blastomeres. It also is the case that PCOS patients showed better clinical and laboratory IVM outcomes compared to the non-PCOS group [35, 36]. Our findings showed no significant differences in the number of oocytes retrieved, rates of in vitro maturation, fertilization, cleavage, and blastocyst formation in PCOS and non-PCOS patients.

For our clinical outcome in IVM/ET cycles, however, the difference between the two groups was non-significant, the non-PCOS group showed better outcomes in the cumulative clinical pregnancy rate (38.5% vs. 35.7%), implantation rate (32.0% vs. 24.1%), biochemical pregnancy (43.2% vs. 42.8%) and miscarriage rate (5.8% vs. 9%) compared to the PCOS group, respectively. The latter group showed a comparable cumulative clinical pregnancy of 35.7% with the outcome of 35.0% for ICSI and IVM cycles in PCOS reported by Walls et al. [15].

Our findings showed a lower outcome efficiency compared with the non-hCG primed IVM cycles reported in the Walls et al. study, which compared the outcome of 98 IVF cycles with 80 cycles of non-hCG primed IVM treatment in PCOS patients. They found that the cumulative clinical pregnancy and implantation rates were nearly 51% and live birth rate was 41.3% [14], which was consistent with other studies that used similar approaches [9, 37]. These high rates of cumulative clinical pregnancy and implantation may be due to the transfer of blastocyst embryos in fresh and frozen ET cycles. In our study, frozen ET cycles of IVM blastocysts yielded nearly the same clinical pregnancy (37.1–47.1%) and implantation rates (28.2–45%) as the Walls report of frozen ET cycles (35.5, 44%) [14].

While our findings were comparable to Zheng’s randomized controlled study that investigated the effect of hCG priming in IVM cycles in 82 PCOS patients who underwent Day3 ET cycles. They found that the rate of in vitro maturation was significantly higher in the hCG-primed group (55.4% vs. 42.2%; *p* < 0.05) than the non-hCG primed group with comparable fertilization rates (63.4% vs. 65.4%) between the groups. There were no significant differences in the clinical pregnancy rate (37.5% vs. 50%), live birth rate (22.5% vs. 30%), and implantation rate (32.8% vs. 32.5%) between the two groups [38].

Our findings showed better clinical pregnancy and implantation rates in frozen ET cycles compared to fresh ET cycles in PCOS and non-PCOS groups. This might be related to the stage of embryo that was transferred, since blastocyst embryos were transferred in frozen ET cycle while cleavage embryos were transferred in fresh ET cycles. According to natural selection, only good quality cleavage embryos have the ability to develop to the blastocyst stage. Thus, this might be related to asynchrony between the state of the endometrium and the IVM embryos.

Additionally, the blastocyst formation rates from IVM oocytes, an indirect measure for early embryo arrest, were comparable in PCOS and non-PCOS patients (28% vs 28.2%, respectively) while the blastocyst formation rate from in vivo MII oocytes were 57.1% vs 62.5, respectively, as shown in Table 2. However, the size of in vivo MII oocytes that were retrieved was less than 12 mm in diameter, but the blastocyst formation rate was still higher than that of IVM oocytes. However, Walls reported that the blastocyst formation rate in PCOS-IVM embryos was comparatively high (48.2%) [15]. Unfortunately, it was associated with an increased rate of early embryo arrest during the third cytokinesis (Day 3–4 stage) compared to the ICSI group [15]. Altogether these findings suggested that the IVM itself is the possible cause of early embryo arrest rather than the PCOS condition. In conclusion, a better understanding and improvement of IVM procedure is still needed.

The variety of factors that increased the possibility of early embryo arrest include genetic causes, oxidative stress, aneuploidy, energy stores defects [19, 39, 40], and mitochondrial dysfunction through ΔΨm that drives the conversion of ADP to ATP by respiratory chain enzymes [41]. Recently, Lei et al. found that cryopreservation significantly decreased the inner mitochondrial membrane potential of vitrified IVM oocytes compared to fresh IVM oocytes (*P* < 0.05) [42]. For the first time, we compared the mitochondrial membrane potential of IVM and IVF oocytes. Using LSCM, we found a significant decrease in the ΔΨm of IVM oocytes compared to control-IVF group (*P* < 0.001) with no difference between PCOS and non-PCOS groups (*P* = 0.7).

Unlike in vivo maturation, which was accompanied by increased cAMP levels, which in turn, induced epidermal growth factor (EGF)-like peptide expression in cumulus cells leading to cumulus expansion and oocyte maturation, in vitro maturation of oocytes is a spontaneous process due to decreased EGF-like peptides that result from a sudden drop in immature COC cAMP [43–46], which might account for the lower mitochondrial function.

However, FSH stimulates and maintains EGF-like peptide expression in vivo, which was unable to do so when added to the IVM media. On the other hand, in animal studies, supplementation with EGF-like peptides significantly increased mitochondrial activity in oocytes matured in vitro through enhanced glucose metabolism and protein glycosylation, which in turn, increased the developmental competence of oocytes and blastocyst formation rate [47, 48]. Also, the pre-maturation stage with cAMP modulators and EGF-like peptides in the IVM technique could attenuate spontaneous oocyte maturation and extend cumulus oocyte gap-junction, resulting in a significant increase in oocyte developmental competence [46, 49, 50].

Based on the above evidence, the IVM-associated high rate of early embryonic arrest might be attributed to mitochondrial dysfunction. Thus, further laboratory investigations with large samples in human are still needed to improve the clinical efficiency of mitochondrial nutrients that can be added to IVM culture medium.

Regarding the consequence of the higher aneuploidy rate in arrested embryos, Previous studies reported that nearly 70% of arrested and slowly cleaving embryos had chromosomal abnormalities, which suggested aneuploidy was a major cause of embryonic arrest [21, 51, 52]. To exclude the possible effect of ovarian hyperstimulation on the aneuploidy rate, Labarta et al. found that moderate ovarian stimulation did not significantly increase the aneuploidy rate [53].

Using FISH, Requena et al. revealed a 60% aneuploidy rate in IVM embryos compared to 33% in control groups [54]. While, Benkhalifa et al. found a 32.6–49.2% aneuploidy rate in IVM arrested embryos from PCOS patients [55]. Although the incidence of aneuploidy greatly varies in the two studies, the smaller number of chromosomes screened, a main limitation of FISH, different IVM protocols that were applied, and variations between participants (normal oocyte donors and PCOS women) may account for the observed differences. Recently, using LSCM, several studies reported that in vitro culture had no impact on the IVM efficiency or the frequency of meiotic anomalies in POCS patients compared to controls, but they reported an increase in the percent of meiotic anomalies, which suggested that IVM itself might cause an increase in the incidence of meiotic abnormalities that then resulted in chromosomal disorders [56, 57].

We investigated the aneuploidy rates in arrested (IVM and IVF) embryos using NGS on small DNA fragments that covers the whole chromosomes [30]. The sequencing results showed that the aneuploidy rates were comparable (75, 75, and 66.6%) in IVM-PCOS, IVM-non PCOS, and control IVF arrested embryos, respectively. Our results were comparable to Zhang’s retrospective study on chromosomal abnormalities in IVM and IVF embryos (58.7% vs. 57.4%, respectively) [58]. Therefore, aneuploidy does not appear to be the predisposing reason for the high incidence of early embryo arrest in IVM cycles.

## Conclusions

Mild stimulated IVF/M cycles can be an attractive choice for normal ovulatory and anovulatory PCOS patients not only to avoid OHSS but also due to its simplified patient-friendly ART procedure that has lower cost and acceptable clinical pregnancy rates. The current study reported no significant differences in the laboratory and clinical outcomes of the IVM procedure in PCOS and non-PCOS patients, which encourage clinicians to apply such technique for non-PCOS as well PCOS patients. In addition, frozen and thawed ET cycles had a higher clinical pregnancy and implantation rate compared to that of fresh ET cycles. Thus, it is necessary to pay more attention to the possibility of asynchrony between the endometrium and the embryos.

Additionally, we found that embryo arrest is IVM procedure-related rather than due to the PCOS condition itself. As seen with using LSCM, MII oocytes after IVM culture showed decreased mitochondrial membrane potentials compared to in-vivo MII oocytes, but with no difference between PCOS and non-PCOS patients. NGS showed comparable aneuploidy rates of arrested IVM/IVF embryos. Therefore, we concluded that IVM itself adversely affected oocyte and embryo development by affecting mitochondrial function that seemed to be the possible cause for lower blastocyst formation rates in IVM cycles. Further laboratory investigations with larger sample sizes are still needed to improve IVM oocyte and embryo competence in vitro culturing.

## Data Availability

Not applicable.
